# Supplementary material for: Loss of histone methyltransferase SETD1B in oogenesis results in the redistribution of genomic histone 3 lysine 4 trimethylation
Source: Nucleic Acids Res. 2022 Feb 7;50(4):1993–2004. doi: 10.1093/nar/gkac051 (PMC8887468; doi:10.1093/nar/gkac051)

Supplementary Item Legends

Supplementary Table 1. Raw and uniquely mapped read depths are provided for all sequencing libraries generated for this study. PBAT: post-bisulphite adaptor tagging

Supplementary Table 2. Correlation matrix of R-values for all replicates of single cell RNA-seq. All oocyte transcripts were quantitated using log2RPM.

Supplementary Figure 1. (A) Hierarchical clustering for H3K4me3 ChIP-seq replicates in *Setd1b* WT (N=3) and *Setd1b* cKO (N=3) GV oocytes, and 10% input replicates (N=4). Clustering was done using 5kb running windows and enrichment normalised RPKM. **(B)** The screenshot shows the normalised enrichment for H3K4me3 for 1kb running windows with a 500bp step in the *Setd1b* cKO and WT GV oocyte replicates. Significant differentially enriched 5kb windows are shown in the *Setd1b* gain/loss track, with red and blue bars showing windows that gain and lose H3K4me3 in *Setd1b* cKO, respectively. **(C)** Top: pie charts show the proportion of 5kb windows that gain (left) or lose (middle) H3K4me3 in the *Setd1b* cKO GV oocytes that overlap transcription start sites (TSSs) or genic regions, compared to a random set (right) of 5kb windows ($p<0.0001$ and $p<0.0001$, respectively, Chi-square). Bottom: pie charts show the proportion of the inter-genic 5kb windows that gain (left) or lose (middle) H3K4me3 in the *Setd1b* cKO GV oocytes that overlap combinatorial peaks for histone modifications in GV oocytes (Hanna et al., 2018), compared to the inter-genic random (right) 5kb windows ($p<0.0001$ and $p<0.0001$, respectively, Chi-square). **(D)** The scatterplot shows enrichment for H3K27ac and H3K27me3 for 5kb running windows (normalised RPKM) in d25 GV oocytes. The windows that gain and lose H3K4me3 in the *Setd1b* cKO are highlighted in red and blue, respectively. **(E)** The barplot shows the relative enrichment for dimer content in regions that gain H3K4me3 in the *Setd1b* cKO compared to a random set of 5kb probes.

Supplementary Figure 2. (A) The heatmap shows DEGs in the *Setd1b* cKO and WT GV oocyte replicates, quantitated as per gene-normalised log_2RPKM. **(B)** The boxplot shows the log_2 fold change in gene expression between the *Setd1b* cKO and WT for TSSs that overlap regions that gain (N=415), lose (N=914) or were unchanged (N= 26,428) for H3K4me3 in the *Setd1b* cKO GV oocytes. Pair-wise comparisons between gain and unchanged and loss and unchanged were done with two-tailed t-test (p=7.3E-8 and p=1.4E-14, respectively). **(C)** Principal component analysis shows scRNA-seq replicates for *Setd1b* cKO GV (N=4), *Setd1b* WT GV (N=3), *Setd1b* cKO MII (N=4), and *Setd1b* WT MII (N=4) oocytes. Expression of oocyte transcripts were quantitated using log_2RPM. **(D)** The scatterplot shows log_2 fold change (cKO/WT) in gene expression (RPKM) for *Setd1b* cKO DEGs in *Setd1b* cKO GV and MII oocytes. **(E)** The barplot shows the average gene expression for H3K4 methyltransferases (*Setd1a*, *Mll1*, *Mll2*, *Mll3*, *Mll4*), H3K4 methyltransferase complex subunits (*Cxxc1*, *Rbbp5*, *Ash2l*, *Wdr5*, *Dpy30*), and *de novo* DNA methyltransferases (*Dnmt3a*, *Dnmt3b*, *Dnmt3l*) in *Setd1b* cKO and WT GV oocytes. The whiskers show standard deviation between biological replicates, while the asterisks shows those genes that are significant DEGs.

Supplementary Figure 3. (A) The Venn diagrams show the overlap between genes upregulated in oogenesis and up- and downregulated DEGs in *Setd1b* cKO GV oocytes. **(B)** The Venn diagrams show the overlap between genes upregulated in oogenesis and up- and downregulated DEGs in *Cxxc1* cKO GV oocytes. **(C)** The Venn diagrams show the overlap between up- and downregulated DEGs in the *Cxxc1* cKO GV oocytes with up- and downregulated DEGs in *Setd1b* cKO GV oocytes. **(D)** The scatterplot shows the log_2 fold change (cKO/WT) in gene expression (RPKM) for *Setd1b* cKO DEGs in the *Setd1b* cKO GV oocytes compared to the *Cxxc1* cKO GV oocytes. **(E)** The scatterplot shows the log_2 fold change (cKO/WT) in gene expression (RPKM) for *Setd1b* cKO DEGs in the *Setd1b* cKO GV oocytes compared to the *Mll2* cKO GV oocytes.

Supplementary Figure 4. (A) The scatterplot shows average normalised enrichment for H3K4me3 for 5kb running windows (N=544,879) between *Cxxc1* cKO (N=2) and WT (N=2) GV oocytes. Differentially enriched windows identified in *Setd1b* cKO are shown in blue (loss) and red (gain). **(B)** The screenshot shows the normalised enrichment for H3K4me3 for 1kb running windows with a 500bp step in *Setd1b*

cKO, *Setd1b* WT, *Cxxc1* cKO and *Cxxc1* WT GV oocytes. Significant differentially enriched 5kb windows are shown in the *Setd1b* gain/loss track, with red and blue bars showing windows that gain and lose H3K4me3 in *Setd1b* cKO, respectively. **(C)** The boxplot shows the H3K4me3 enrichment (normalised RPKM) in d5 non-growing oocytes (NGO), d10 growing oocyte (GO), d15 GV, d25 GV, *Setd1b* WT, *Setd1b* cKO, *Cxxc1* WT and *Cxxc1* cKO GV oocytes for 5kb windows that significantly gain (left) or lose (right) H3K4me3 in *Setd1b* cKO oocytes.

Supplementary Figure 5. (A) Hierarchical clustering for replicates of DNA methylation in *Setd1b* WT, *Setd1b* cKO, *Cxxc1* WT and *Cxxc1* cKO GV oocytes. Clustering was done using 100-CpG windows (excluding the Y chromosome) with a minimum coverage of 10-CpGs. **(B)** The scatterplot shows the DNA methylation in *Cxxc1* cKO and WT GV oocytes across 100-CpG windows, with at least 10 informative CpGs in grouped replicates. *Setd1b* hypomethylated (green) and hypermethylated (purple) differentially methylated regions (DMRs) are highlighted. **(C)** The screenshot shows DNA methylation of 100-CpG windows with at least 10 informative CpGs per replicate for *Setd1b* cKO and WT oocytes and per grouped replicate for *Cxxc1* cKO and WT GV oocytes. Regions that gain or lose DNA methylation (*Setd1b* cKO DMRs) are shown in the labelled annotation tracks as purple and green bars, respectively.

Supplementary Tables

Supplementary Table 1

Sample	Library	Raw Reads	Uniquely mapped
Setd1b WT rep1	H3K4me3 ChIP-seq	20,014,719	12,726,842
Setd1b WT rep2	H3K4me3 ChIP-seq	40,103,864	10,579,220
Setd1b WT rep4	H3K4me3 ChIP-seq	26,368,689	15,942,467
Setd1b cKO rep1	H3K4me3 ChIP-seq	26,790,166	12,427,601
Setd1b cKO rep3	H3K4me3 ChIP-seq	38,267,203	11,611,307
Setd1b cKO rep4	H3K4me3 ChIP-seq	36,404,048	14,138,852
Setd1b WT rep1	PBAT	65,496,468	29,075,293
Setd1b WT rep2	PBAT	49,773,592	25,975,398
Setd1b WT rep3	PBAT	60,201,429	32,757,844
Setd1b cKO rep1	PBAT	69,046,033	29,903,629
Setd1b cKO rep2	PBAT	63,538,005	33,224,984
Setd1b cKO rep3	PBAT	68,637,891	34,873,524
Setd1b WT rep1	scRNA-seq	16,915,059	9,329,696
Setd1b WT rep2	scRNA-seq	17,712,949	9,726,867
Setd1b WT rep3	scRNA-seq	14,765,556	8,006,481
Setd1b cKO rep1	scRNA-seq	13,555,016	7,138,433
Setd1b cKO rep2	scRNA-seq	16,236,758	8,997,586
Setd1b cKO rep3	scRNA-seq	15,506,713	8,462,170
Setd1b cKO rep4	scRNA-seq	11,803,207	6,063,243

Supplementary Table 2

	Setd1b cKO 1	Setd1b cKO 2	Setd1b cKO 3	Setd1b cKO 4	Setd1b WT 1	Setd1b WT 2	Setd1b WT 3
**Setd1b cKO 1**	1.000	0.958	0.952	0.941	0.941	0.938	0.936
**Setd1b cKO 2**	0.958	1.000	0.957	0.930	0.945	0.941	0.929
**Setd1b cKO 3**	0.952	0.957	1.000	0.938	0.937	0.935	0.931
**Setd1b cKO 4**	0.941	0.930	0.938	1.000	0.916	0.917	0.936
**Setd1b WT 1**	0.941	0.945	0.937	0.916	1.000	0.960	0.948
**Setd1b WT 2**	0.938	0.941	0.935	0.917	0.960	1.000	0.948
**Setd1b WT 3**	0.936	0.929	0.931	0.936	0.948	0.948	1.000

Supplementary Figure 1

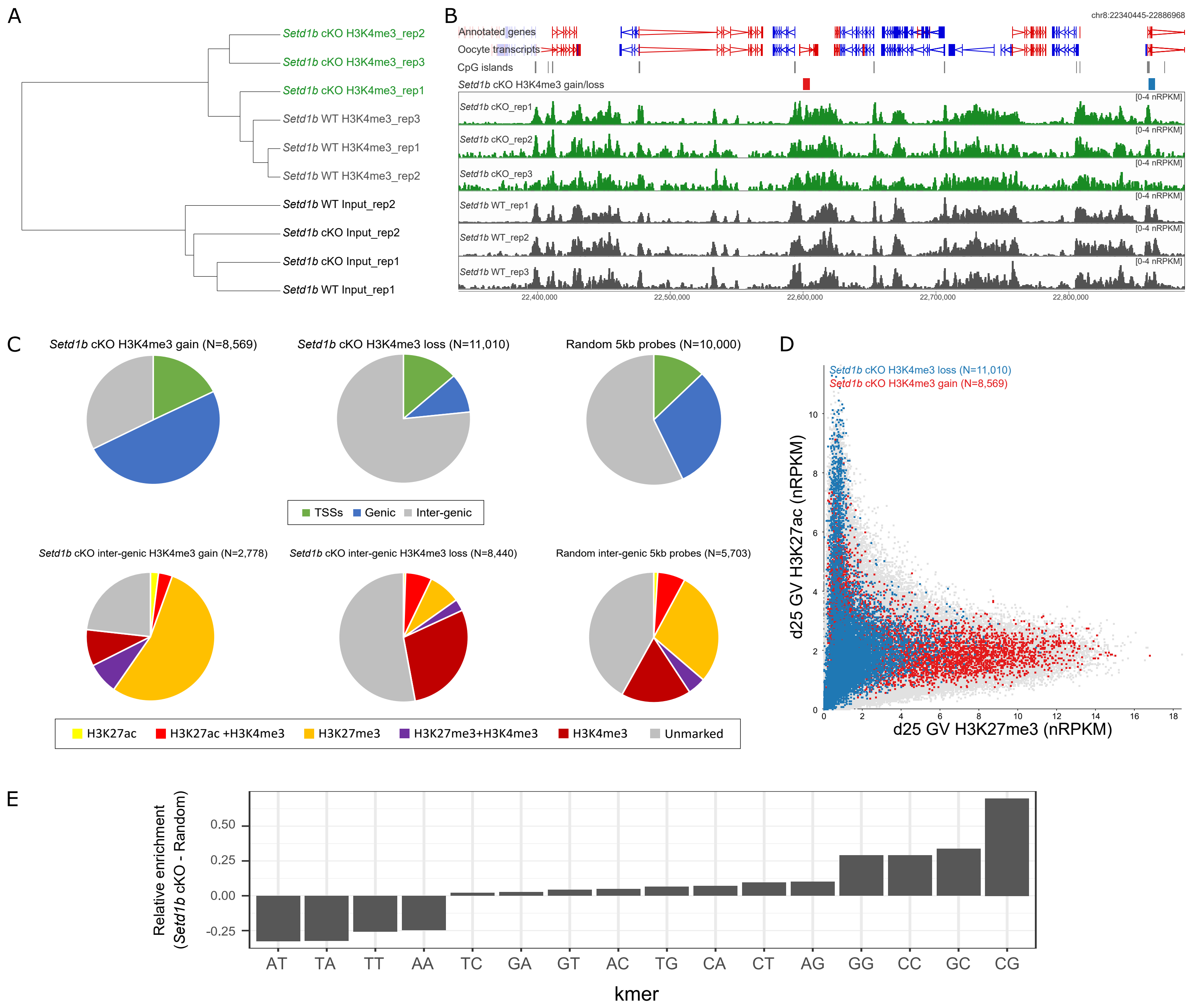

Supplementary Figure 2

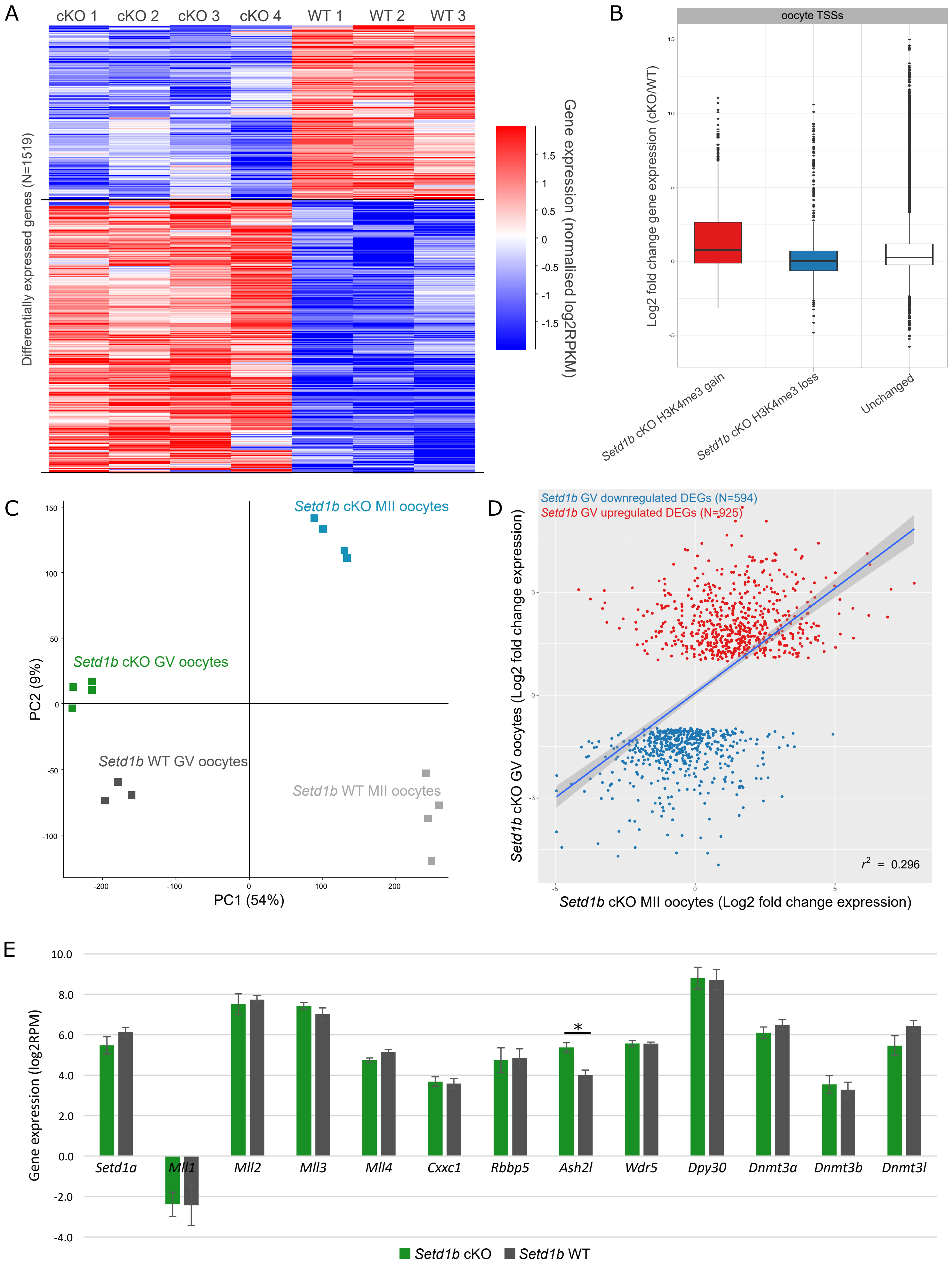

Supplementary Figure 3

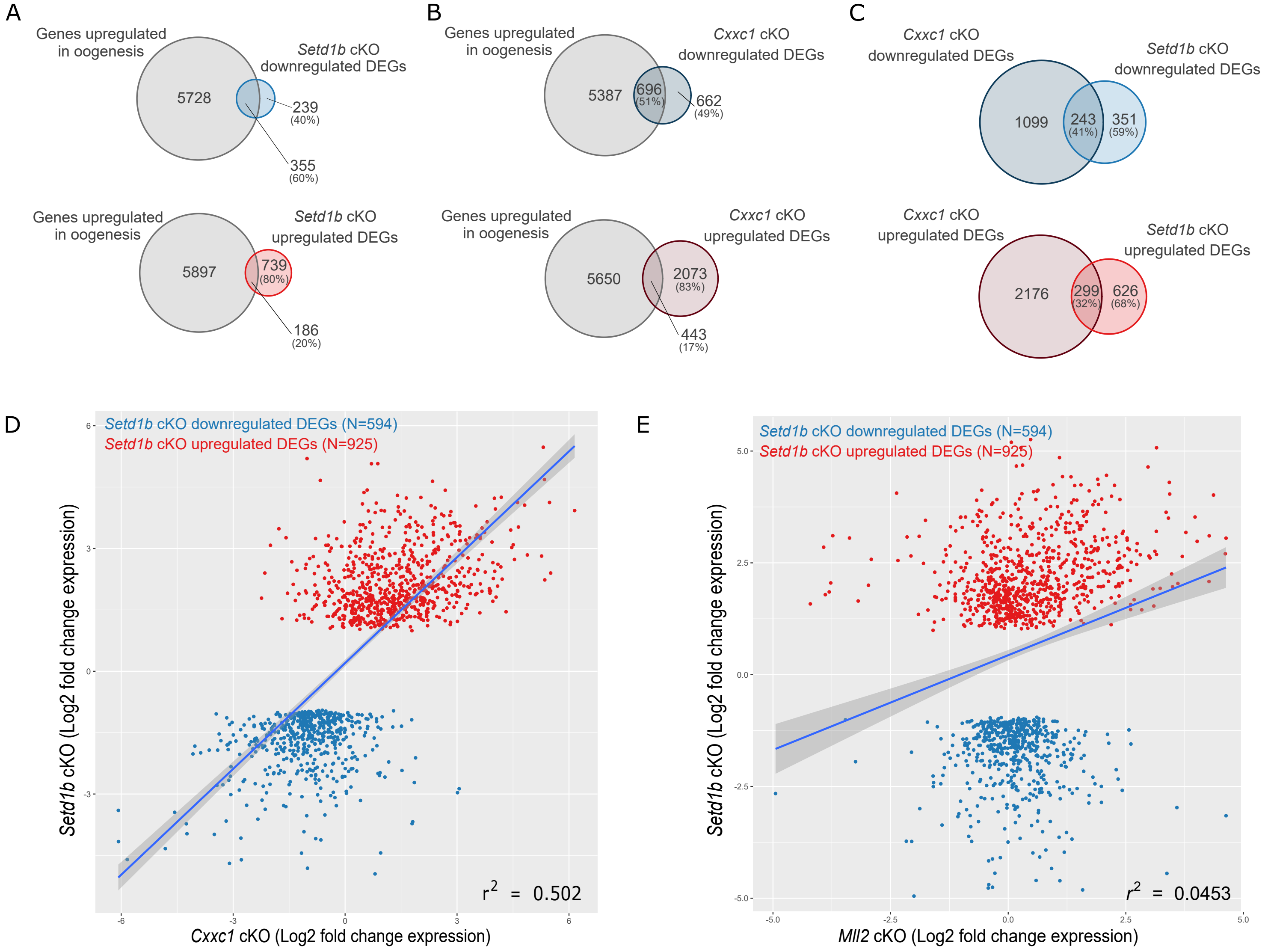

Supplementary Figure 4

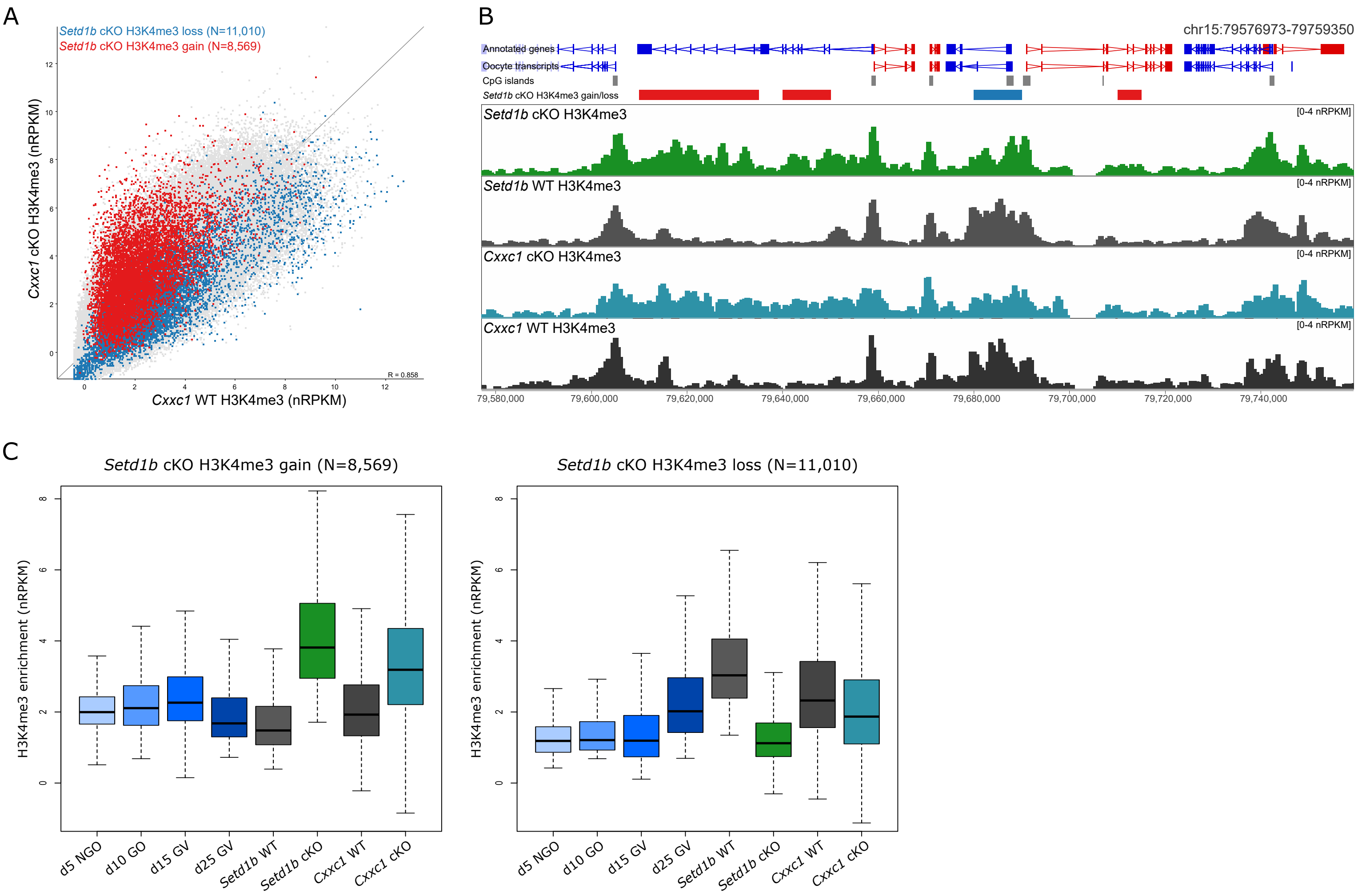

Supplementary Figure 5

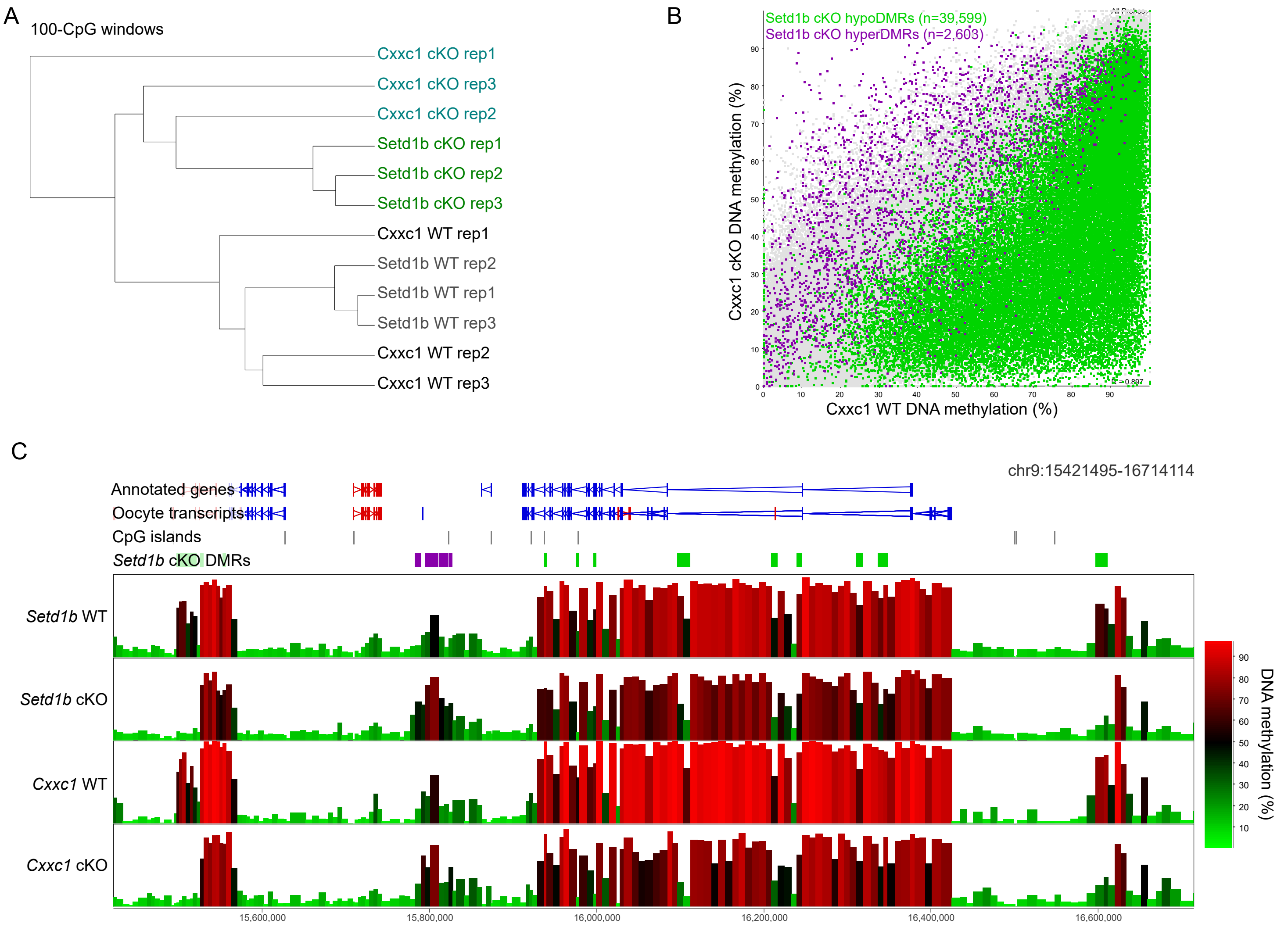

Supplement: gkac051_Supplemental_File [file gkac051_supplemental_file.pdf]
